# An exploration of sex-specific linkage disequilibrium on chromosome X in Caucasians from the COGA study

**DOI:** 10.1186/1471-2156-6-S1-S81

**Published:** 2005-12-30

**Authors:** Miranda E Cox, Joel K Campbell, Carl D Langefeld

**Affiliations:** 1 Section on Biostatistics, Department of Public Health Sciences, Wake Forest University School of Medicine, Winston-Salem, North Carolina, USA

## Abstract

This paper explores the decay of linkage disequilibrium (LD) on the autosomes and chromosome X. The extent of marker-marker LD is important for both linkage and association studies. The analysis of the Caucasian sample from the Collaborative Study on the Genetics of Alcoholism study revealed the expected negative relationship between the magnitude of the marker-marker LD and distance (cM), with the male and female subgroups exhibiting similar patterns of LD. The observed extent of LD in females was less across the pseudoautosomal markers relative to the heterosomal region of chromosome X. Marked differences in LD patterns were also observed between chromosomes X and the 22 autosomes in both males and females.

## Background

In the human genome, alleles at two loci on the same chromosome often show stochastic dependence. This nonrandom pair-wise allelic association, or linkage disequilibrium (LD), is both interesting evolutionarily in its own right and a powerful tool in the mapping of genes for complex genetic traits. The development of the technology to genotype large numbers of single-nucleotide polymorphisms (SNPs) provides the means to explore the extent and patterns of linkage disequilibrium in the genome. The two pseudoautosomal regions on chromosomes X and Y (PAR1 and PAR2) are approximately 2.6 and 0.4 Mb respectively, and are located at the tips of the sex chromosomes. Within these regions pairing and recombination may occur between X and Y as it naturally does in the autosomes or between two X chromosomes in females. Dense genetic linkage maps have revealed a high rate of recombination in PAR1 [1], prompting comparisons between LD patterns in PAR1 and the heterosomal region on chromosome X. The purpose of this paper is to explore the rate of decay in LD as a function of distance between males and females both across and within autosomal and sex chromosomes.

## Methods

Data used for our analyses were obtained from the Collaborative Study on the Genetics of Alcoholism (COGA). COGA is a large-scale family study designed to identify genes that contribute to the risk for alcoholism. Our analyses used data genotyped by both Affymetrix and Illumina. Four hundred and thirty-four genetic markers on chromosome X were analyzed (310 SNPs by Affymetrix and 124 SNPs by Illumina). SNPs genotyped and analyzed on the 22 autosomes by Affymetrix and Illumina totaled 15,371. Given that the proportion of self-reported non-Caucasian individuals in the COGA sample was small, our results are based on 102 Caucasian pedigrees. These 102 pedigrees were composed of 430 genotyped male and 446 genotyped female participants; 9 genotyped participants whose sex was not reported were not included in these analyses.

A sample of 172 genotyped unrelated participants was derived from the COGA pedigrees in order to compute LD statistics. To form the sample of unrelateds, a hierarchical approach was used. In pedigrees in which founders were genotyped, all available founders were chosen for analysis. Where founders were not genotyped, the proband (index case) was used if a proband was declared. Otherwise, the individual with the smallest COGA identification number within each family who had been genotyped was chosen. SNPs with minor alleles <0.05 were removed from analysis. The statistic *r*^2 ^= D^2^/(p_1_p_2_q_1_q_2_) was calculated for each adjacent pair of markers across the genome. Here, D is defined as D = x_11 _- p_1_q_1 _(x_11 _is the frequency of haplotype A_1_B_1_, and p_1 _and q_1 _are the frequencies of alleles A_1 _and B_1 _at loci A and B, respectively) [2]. In males phase is known on the heterosomal region of chromosome X. Thus, in males estimates were computed directly from the genotype data using maximum likelihood estimation. For females, we used the software Dprime (available from authors) which employs the EM (expectation maximization) algorithm, a maximum likelihood method, for haplotype frequency estimation [3]. For adjacent SNPs on all chromosomes, we explored the fit of the *r*^2 ^results to the exponential, Weibull, Gamma and log-normal distributions, adjusting for distance (cM). Among these distributions, the Weibull distribution provided the best fit and will be reported. Inferences based on the other distributions are comparable. Note that these models do not account for the fact that each individual contributes multiple observations to the analysis (i.e., once for each marker pair.) Chi-squared tests were used to compare the sex-specific Weibull distributions of *r*^2 ^1) within the X chromosome, 2) between the pter pseudoautosomal region (PAR1) on X and the heterosomal region on X, 3) between PAR1 and the autosomes, and 4) between chromosome X and the autosomes.

## Results

### Autosomes

Sex-specific LD analyses across the genome revealed the anticipated rapid decay of LD with increased distance (cM) for both males and females (-coefficients for distance are  = -0.1005,  = -0.6457, respectively). Very few adjacent SNP markers exhibited significant LD (*r*^2 ^> 0.7), generally supporting the use of these markers in linkage analyses.

### Chromosome X

As observed in the autosomal analysis, most of the SNPs in close proximity (<0.1 Mb apart) on chromosome X did not exhibit significant LD (*r*^2 ^> 0.7). A comparison of the *r*^2 ^measures for adjacent SNPs showed that the LD distribution for males and females on chromosome X were comparable (males were coded as 0 and females coded as 1, *p *= 0.9573) (Table 1A).

**Figure 1:**
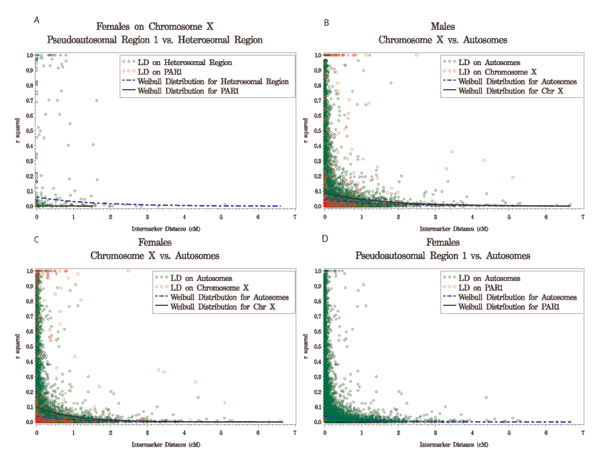


Only females were used to compare the pseudoautosomal region PAR1 to the heterosomal region of chromosome X (chromosome Y data not available). The first 7 SNPs lie in the PAR1 region, while 265 SNPs lie in the heterosomal region of X (46–130 cM). PAR2 was not analyzed due to lack of SNPs in that region. The 7 PAR1 SNPs exhibited very low LD (*r*^2 ^< 0.1). The chi-squared test showed a significant difference in LD for the two regions on X (heterosomal markers were coded as 0 and PAR1 coded as 1, *p *= 0.0045) (Table 1B and Figure 1A).

Although the autosomes and chromosome X both showed a similar decline in sex-specific LD as intermarker distance increased, the rate of decay proved to be quite different. LD between adjacent SNPs on chromosome X tended to be concentrated more toward the extremes (either very strong LD (*r*^2 ^> 0.9) or very weak LD (*r*^2 ^< 0.1)), while LD in the autosomes decreased gradually with distance (Tables 1C, and Table 1D, and Figure 1B and Figure 1C). Chi-squared tests confirmed this difference in both males and females (autosomes were coded as 0 and chromosome X coded as 1, p < 0.0001 for both comparisons). The chi-squared test also revealed a difference in LD between PAR1 and the autosomes (*p *= 0.0019) for females (Table 1E, Figure 1D).

## Discussion

The primary results from this study are the observed variations in recombination rates between the autosomes and chromosome X and between PAR1 and the heterosomal regions of chromosome X. These results have obvious analysis implications. Thorough fine mapping of regions with high recombination rates requires significantly more SNPs to delineate fully the LD block structure and capture the haplotypic variation. Recombination of chromosomes tends to increase genetic diversity. More specifically, recombination is not the source of new genes but the source of new combinations of genes. The evolutionary significance as to why PAR1 and the heterosomal region on chromosome X vary in the extent of LD compared to each other and to the autosomes is not immediately obvious. That is, it is not clear whether the observed patterns in these genomic regions are the results of current or past selective pressure on specific genes, or represent an interesting characteristic of the genome void of meaningful evolutionary cause or significance. The resolution of this question likely must wait until the functions of the individual genes in these regions are known and placed in the context of modern evolutionary theory.

A significant limitation of this investigation is that the SNPs are not dense enough to appropriately explore differences in block structures, particularly in the PAR1 region. A more dense set of markers across the genome would provide additional information about LD structures as well as potential "recombinational hot-spots". Furthermore, if sufficient data from pseudoautosomal region 2 were available, a comparison of LD in PAR1 and PAR2 would be of interest. With the advent of genome-wide association analysis using 500 K chips, these questions can be explored in detail.

## Conclusion

A comparison of sex-specific LD across chromosome X revealed a comparable rate of decay in LD with increased distance for males and females. When compared with the autosomes, however, the rate decay of LD on chromosome X was significantly more rapid. In addition, we observed much lower LD on pseudoautosomal region 1 (PAR1) of chromosome X compared with the heterosomal region of chromosome X. This observation of low LD across PAR1 is consistent with previous results that suggest higher recombination rates on PAR1 when compared with the heterosomal region [4].

## Abbreviations

COGA: Collaborative Study on the Genetics of Alcoholism

EM: Expectation maximization

LD: Linkage disequilibrium

SNP: Single-nucleotide polymorphism

## Authors' contributions

MEC, JKC, and CDL contributed to the formulation of the question and the writing of the manuscript. MEC and JKC conducted the analyses.

## Figures and Tables

**Table 1:** Comparisons of sex-specific distributions of linkage disequilibrium.

	Intercept	*Sex^a^	Region^a^	Distance (cM)	Scale	Shape
A. Chromosome X-Females vs. Males						

	-2.0340	-0.0092	-^b^	-0.2799	2.1870	0.4573
SE()	0.1375	0.1725	-	0.1025	0.0684	0.0143
χ^2^	218.82	0	-	7.45	-	-
*P*	<0.0001	0.9573	-	0.0063	-	-

B. Chromosome X-PAR1 vs. Heterosomal						

	-1.9951	-	-3.2261	-0.6541	2.2447	0.4455
SE()	0.1899	-	1.1347	0.2532	0.1218	0.0242
χ^2^	110.4	-	8.08	6.68	-	-
*P*	<0.0001	-	0.0045	0.0098	-	-

C. Females-Chromosome X vs. Autosomes						

	-1.5356	-	-0.9522	-1.0045	2.1099	0.4740
SE()	0.1228	-	0.1235	0.0177	0.0134	0.0030
χ^2^	156.40	-	59.45	3210.72	-	-
*P*	<0.0001	-	<0.0001	<0.0001	-	-

D. Males-Chromosome X vs. Autosomes						

	-1.7596	-	-0.5890	-0.6457	1.9684	0.5080
SE()	0.1065	-	0.1074	0.0149	0.0125	0.0032
χ^2^	273.2	-	30.09	1872.87	-	-
*p*	<0.0001	-	<0.0001	<0.0001	-	-

E. Females-PAR1 vs. Autosomes						

	-5.1301	-	2.6487	-1.0247	2.1055	0.4749
SE()	1.0528	-	1.0529	0.0172	0.0135	0.0030
χ^2^	23.75	-	6.33	3552.71	-	-
*p*	<0.0001	-	0.0119	<0.0001	-	-

^a^For the comparison of sex on chromosome X, males were coded as zero and females coded as 1. For the (females-only) comparison of regions on chromosome X, the heterosomal markers were coded as 0 and PAR1 coded as 1. For the sex-specific comparisons of chromosome X to the autosomes, the autosomes were coded as 0 and chromosome X coded as 1.

^b^-, not done.
